# Impact of mobile health-enhanced supportive supervision and supply chain management on appropriate integrated community case management of malaria, diarrhoea, and pneumonia in children 2-59 months: A cluster randomised trial in Eastern Province, Zambia

**DOI:** 10.7189/jogh.10.010425

**Published:** 2020-06

**Authors:** Godfrey Biemba, Boniface Chiluba, Kojo Yeboah-Antwi, Vichael Silavwe, Karsten Lunze, Rodgers K Mwale, Davidson H Hamer, William B MacLeod

**Affiliations:** 1 National Health Research Authority, Lusaka, Zambia; 2 Department of Global Health, Boston University School of Public Health, Boston, Massachusetts, USA; 3 Zambian Center for Applied Health Research and Development (ZCAHRD), Lusaka, Zambia; 4 Ministry of Health, Child Health Unit, Lusaka, Zambia; 5 Division of Internal Medicine, Department of Medicine, Boston University School of Medicine and Boston Medical Centre, Massachusetts, USA.; 6 United Nations Children’s Fund (UNICEF), Lusaka, Zambia; 7 Section of Infectious Diseases, Department of Medicine, Boston University School of Medicine, Boston, Massachusetts, USA

## Abstract

**Background:**

Despite progress made over the past twenty years, child mortality remains high, with 5.3 million children under five years having died in 2018 globally. Pneumonia, diarrhoea, and malaria remain among the commonest causes of under-five mortality; contributing 15%, 8%, and 5% of global mortality respectively. Recent evidence shows that integrated community case management (iCCM) of pneumonia, diarrhoea, and malaria can reduce under-five mortality. However, despite growing evidence of the effectiveness of iCCM, there are implementation challenges, especially stock out of iCCM commodities and inadequate supportive supervision of community health workers (CHWs). This study aimed to address these two key challenges to successful iCCM implementation by using mobile health (mHealth) technology.

**Methods:**

This cluster randomised controlled trial compared health centre catchment areas (clusters) where CHWs and their supervisors implemented mHealth-enhanced iCCM supportive supervision and supply chain management vs clusters implementing iCCM as per current Zambian guidelines. CHWs in intervention clusters used community DHIS2 platform on mobile phones to report on a weekly basis children with iCCM conditions and make requisitions for iCCM commodities. Their supervisors received electronic reports on disease caseloads and monthly automated supervision reminders. The supervisors on receipt of requisitions, organized the medical supplies and notified CHWs for collection. Intention-to-treat analysis on the primary outcome, the percentage of children aged 2-59 months receiving appropriate treatment for malaria, pneumonia, or diarrhoea from an iCCM trained CHW, was performed using a generalized linear model. Prevalence ratios and 95% confidence intervals comparing the prevalence of appropriate treatment in the intervention and control groups were calculated using log binomial regression with an exchangeable correlation matrix, adjusted for clustering by health facility.

**Results:**

In the intervention clusters, 61.3% (98/160) of expected monthly supervision visits took place vs 52.0% (78/150) in the controls. A total of 3690 children 2-59 months old presented with malaria, diarrhoea, or pneumonia. In the intervention group, 65.9% (1,252/1,899) of children received appropriate care for iCCM conditions, compared to 63.3% (1,134/1,791) in the control group. The mHealth intervention was associated with 18.0% improvement in supportive supervision and 21.0% increase in appropriate treatment for pneumonia; these changes were not statistically significant. There was a 2-3-fold increase in the proportion of CHWs receiving supplies ordered: prevalence ratios ranged from 2.82 (confidence interval (CI) = 1.50, 5.30) to 3.01 (95% CI = 1.29, 7.00) depending on the particular commodity.

**Conclusion:**

This study was unable to determine whether using mHealth technology would strengthen supervision and supply chain management of iCCM commodities for community-level workers. There was no statistically significant effect of mHealth enhanced iCCM on appropriate diagnosis and treatment for children with malaria, pneumonia, and diarrhoea in rural Zambia. Longer term longitudinal studies are required to determine the impact of mHealth enhanced iCCM on health outputs and outcomes.

**Trial registration:**

ClinicalTrials.gov, NCT02866097

Globally, child mortality remains high; with an estimated 5.3 million deaths of children under 5 years in 2018; 3.3 million of these deaths were from sub-Saharan Africa [1]. Close to a third of all under-five deaths globally are due to pneumonia (15%), diarrhoea (8%), and malaria (5%) [1].

In resource-limited countries, access to health facilities for prompt and appropriate management of these diseases is often complicated by shortages of essential medicines and insufficient human resources. iCCM has been increasingly adopted as a comprehensive, equity-based strategy that complements and extends the reach of public health services by providing timely and effective treatment of malaria, pneumonia and diarrhoea to populations with limited access to facility-based health care providers, and especially children under five years of age [2,3]. In June 2012, the World Health Organization (WHO) and the United Nations International Children’s Education Fund (UNICEF) issued a joint statement supporting iCCM to improve access for the poorest, most underserved children to treatment for these diseases [4].

As part of iCCM, CHWs are trained and supplied with appropriate medicines and diagnostic tools, and receive supportive supervision to correctly diagnose children presenting with fever and treat them for malaria, pneumonia and diarrhoea, using artemisinin-based combination therapy (ACT), oral antibiotics, oral rehydration salts and zinc. The iCCM approach incorporates WHO’s recommendation that all suspected malaria cases undergo diagnostic testing prior to treatment [5]. Following a diagnostic algorithm, all patients are screened for the three diseases and treatment is administered based on the results of the examination and diagnostic testing that includes malaria rapid diagnostic tests (mRDTs), disease history and respiratory rate timers. The availability of high-quality mRDTs has made testing for malaria at the community level possible [6].

The iCCM strategy, especially when mRDTs are used properly, offers several benefits, including early access to appropriate treatment for malaria and pneumonia [7,8], reduction in the inappropriate use of expensive antimalarial drugs [8,9], increased care seeking in the community through increased caretaker trust in adequate treatment from CHWs [10,11], and reductions in health centre attendance of uncomplicated cases that help to reduce the workload at primary health care centres (task-shifting) [11,12].

Despite growing evidence of the effectiveness of iCCM, there are gaps in knowledge on optimal implementation approaches. One major challenge to the effectiveness of iCCM is the frequent stockout of drugs and commodities such as mRDTs [13]. In addition, supportive supervision of CHWs is an important bottleneck to effective iCCM implementation. Insufficient supervision adversely impacts quality of care as well as health worker motivation and retention [14,15]. A recent pilot evaluation of the community health assistant (CHA) strategy in two districts of Zambia highlighted the importance of the availability of drugs and supplies and supervision to improving access to treatment of malaria, diarrhoea, and pneumonia in children under five years [16].

Diarrhoea, malaria and pneumonia are among the commonest causes of morbidity and mortality in Zambia. Overall, 2% of children under age 5 showed symptoms of an acute respiratory tract infection (ARI), 16% exhibited fever, and 15% experienced diarrhoea in the 2 weeks preceding the survey [17]. Malaria remains endemic in all ten provinces, with the entire population considered to be at risk. Health Management Information System (HMIS) data show a steady increase in annual confirmed malaria cases from 2 325 858 in 2011 to 5 503 010 in 2017 [18].

In order to deal with the challenge of these three diseases (diarrhoea, malaria, and pneumonia) Zambia has been implementing the integrated management of childhood illness (IMCI). There are two components to the strategy: facility IMCI and community IMCI (cIMCI). The latter is also called iCCM of diarrhoea, malaria, and pneumonia. iCCM is implemented by trained CHWs who diagnose and treat diarrhoea with zinc and oral rehydration salts (ORS), malaria with artemether-lumefantrine, and pneumonia with oral amoxicillin. The program started in May 2010 in one district and has been scaled to 78 districts and so far, 6724 CHWs and 1114 supervisors have been trained in iCCM.

In a recent Child Health and Nutrition Research Initiative (CHNRI) exercise, experts both at headquarter level and in the field identified 20 priority research questions for iCCM of which strategies to improve supervision, motivation and retention of health workers were ranked highly [19]. Mobile health (mHealth) innovations could offer potential solutions to redirect the immediate monitoring of stocks, at least in part, to CHWs and away from central procurement systems. mHealth also has potential to improve the frequency and quality of supervision and mentoring [20-22]. This potential, however, has not been fully investigated to assess its impact on health outputs and outcomes. Several studies have been conducted on mHealth technology and iCCM implementation. For example, a study in Malawi evaluated the effect of mHealth technology to improve quality of iCCM care delivered by CHWs called Health Surveillance Assistants (HSAs). Although the study found that HSAs using mHealth technology tended to adhere better to the iCCM guidelines than those using paper-based forms, there was no impact on treatment. The authors therefore recommended more studies on mHealth to improve community-level quality of care [23]. Another study conducted in Uganda assessed the impact of mobile phone deployment on iCCM effectiveness in terms of appropriate treatment for diarhhoea, malaria and diarrhoea [24]. The study reported that CHWs supported by mobile phones instituted ‘appropriate’ treatment in 97.1% of fever cases, 88.2% of pneumonia cases, and 92.4% of diarrhoea cases, based on the algorithms which are a component of iCCM training. None of the above studies, however, assessed the impact of mHealth technology on supportive supervision and supply chain management as pathways or strategies to improving appropriate treatment for the three iCCM conditions. Inadequate supportive supervision and ineffective supply chain management of iCCM commodities remain key challenges to effective implementation of iCCM [25]. On the other hand, onsite training and supervision of CHWs have been shown to improve clinical practices [26]. The present study was therefore designed to evaluate strategies to improve a) supervision and quality of care using mHealth technology; and b) integration of iCCM logistics (diagnostics and drug supply) at health facility and central levels to the supply system at the community level [27]. The overall aim was to determine whether using mHealth technology to strengthen supervision and supply chain management (commodities reporting and requisition) for community-level workers would result in improved appropriate diagnosis and treatment for children with malaria, pneumonia, and diarrhoea in rural Zambia.

## STUDY DESIGN AND METHODS

### Design

This cluster randomised controlled trial compared the proportion of children aged 2-59 months who received appropriate treatment of malaria, diarrhoea, and pneumonia between clusters where CHWs implemented mHealth enhanced iCCM (intervention) and clusters where CHWs followed current standard practices (control). A cluster was defined as a health centre/post, including all CHWs in its catchment area that were providing iCCM, and all children 2-59 months old who presented with suspected malaria, pneumonia, or diarrhoea to these CHWs.

In the control clusters, CHWs continued to submit paper-based disease aggregation reports and supplies and requisition forms to the health facilities, and then collected their supplies accordingly. In terms of supportive supervision, health centre staff conducted their routine quarterly supportive supervision; CHWs were given children to diagnose while the supervisor observed and asked questions. Based on the supervisor’s observation and responses from the questions asked, the supervisor then completed a paper-based mentorship form and provided CHWs with feedback.

The intervention consisted of a mobile health (mHealth)-enhanced inventory management, supportive supervision and mentorship approach.

### Study sites

This project was implemented in Chipata and Chadiza districts in Eastern Province, Zambia, with populations of 486 500 and 92 300, respectively. Both districts have cellular coverage from all three major network carriers in Zambia and have previously supported mHealth programs, including Project Mwana, a nationwide scale up of a mHealth strategy designed to reduce the time for reporting early infant HIV diagnoses [28].

### Study population, sample size and sampling strategy

We calculated the sample size necessary to show differences between intervention and control clusters, accounting for clustering of patients within health facilities. We estimated that each CHW would treat on average 8 children under the age of 5 years with malaria, pneumonia, or diarrhoea per month, yielding an estimated total of 3840 under-five children with one of these three conditions over a projected 6-month period of data collection. A recent study on the impact of deploying CHAs at community level in Luwingu and Senanga districts of Zambia showed that the percentage of children under five years treated appropriately for malaria, pneumonia or diarrhoea was 60% [16]. We then calculated the sample size accounting for clusters with a power of 80%, a two-sided 5% alpha, and an intracluster correlation coefficient (k) of 0.20. We calculated our sample size to demonstrate differences greater than 15% over a baseline of 60%. Both the baseline estimate of 60% appropriate treatment and the intracluster correlation coefficient of 0.20 were conservative estimates. We estimated that we would use forty clusters (20 in intervention and 20 in control) and collect data from a minimum of 30 CHW treatment episodes of children under five with malaria, pneumonia, or diarrhoea in each cluster. During the course of implementing the project however, we observed that during the first 1-2 months of data collection the study interventions were not fully established and functioning smoothly due to technical difficulties with mobile phone usage. We therefore increased the sample size from 3840 to 4720 under-fives with malaria, pneumonia, or diarrhoea, which would be sufficient to demonstrate a 15% increase in the proportion of children who received appropriate treatment for the three diseases.

Study participants included health facility staff supervising CHWs, iCCM trained CHWs, and children 2-59 months who had been treated for malaria, pneumonia, or diarrhoea and their caregivers. There were 60 health facilities in the two districts. We selected 40 facilities using stratified random sampling, matched on district, facility type and distance from the district health office. We generated 20 random values of 1 (intervention) and 2 (control) to assign facility pairs to intervention and control. If the first facility pair was assigned to intervention, then its pair was assigned to control. Likewise, if the first facility was assigned to control, then its pair was assigned to intervention. We randomised clusters prior to community sensitization and the initiation of mHealth training.

### Interventions

Prior to beginning data collection, 80 CHWs and 40 health facility staff (CHW supervisors) were trained in a basic one-week iCCM training course using a standard MOH training curriculum. The supervisors were trained in iCCM, supervisory and mentorship skills. Forty CHWs and 40 health center staff (supervisors) were participants in the intervention cluster, while there were 39 CHWs (1 dropout) and 40 supervisors in the control cluster. The main intervention was the use of Java-enabled mobile feature phones, loaded with the iCCM community district health management information systems software version 2 (C-DHIS2) Java 2 platform micro edition (J2ME) aggregation and tracker applications [29,30]. The project procured these phones for CHWs and their supervisors, and provided data bundles through a selected mobile network service provider. Following their training in iCCM, both CHWs and their supervisors in the intervention clusters were trained in the C-DHIS2 mobile applications. We designed the iCCM C-DHIS2 mobile application to be adapted into the existing national DHIS2 platform. The C-DHIS2 mobile consists of two separate applications: the DHIS2 J2ME aggregate capture application and DHIS2 J2ME patient tracker application. These applications are used to enhance inventory management by CHWs, patient referrals to health centres and feedback to CHWs, as well as supportive supervision and mentorship of CHWs by health facility staff. The applications were installed on the CHWs’ mobile phones as well as the CHW supervisors’ feature mobile phones. As explained earlier, the application had two progammes; the aggregator and tracker programmes. The aggregator enabled CHWs, on a weekly basis, to not only electronically submit disease cases diagnosed, treated and referred to the health facility for further management, but also submit electronically requisitions and consumption reports of supplies received. At a click of the “send” button the information became available to all that had access passwords as this was a cloud-based application. Supervisors therefore had real-time access to information about diagnosis, treatment, referrals, and consumption of medicines and diagnostic supplies such as RDTs. These weekly electronic reports facilitated supervision and mentorship. The tracker programme registered each child seen with any danger sign with a unique ID. Using the inbuilt clinical information in the application, CHWs were able to make informed decisions on which cases to refer after administering appropriate pre-referral medication. Information on referrals such as name, age, danger signs observed and pre-referral treatment administered became available to the health facility staff before the patient arrived. The exchange of all this electronic information between CHWs and their supervisors facilitated quality supervision and mentorship.

The applications allowed the CHWs to request drugs and diagnostic supplies, refer patients with danger signs to the health facilities, and send weekly reports on the number of under-five children seen with malaria, pneumonia, or diarrhoea. The application also allowed CHWs in the intervention group to receive an SMS reminder on use of the sick child recording form and submission of weekly reports. Supervisors used the applications to provide feedback to CHWs on referral case outcomes and notify CHWs to collect drugs and diagnostic supplies. Monthly automated messages were sent to supervisors reminding them to conduct supervisory visits with CHWs. Supervisors also had access to an internet-based dashboard with all of the CHW reports. Details of the mHealth application functionalities and implementation have been published elsewhere [29].

### Data collection

Sixteen data collectors and four field supervisors received a five-day training in data collection methods, quality control in data collection, orientation to the study protocol and a review of the data collection instruments. The study used five data collection instruments as summarized in Table S1 in the **Online Supplementary Document**.

### Data management and analysis

The primary outcome was the percentage of children aged 2-59 months receiving appropriate treatment for malaria, pneumonia, or diarrhoea from an iCCM trained CHW. Appropriate treatment was defined as the patient receiving appropriate diagnosis and the correct medication for that diagnosis. Secondary outcomes were availability of iCCM medicines and diagnostic supplies, clinical supervision coverage, and correct illness classification by CHWs. Data entry was managed with the use of TeleForms®, a program that allows paper copies of data collection forms to be scanned into a computer database and verified by a data entry specialist. We have used this approach successfully in previous large research projects in Southern Province, Zambia and it has proven useful in managing and verifying large amounts of data [31].

Baseline characteristics, such as age and gender by study arm, potentially related to the outcome were compared between the two groups using tests as appropriate for continuous or categorical variables. Intention-to-treat analysis (ITT) of the primary outcome was performed using a generalized linear model. Prevalence ratios (PR) and their 95% confidence intervals (CI) comparing the prevalence of appropriate treatment in the intervention and control groups were calculated using log binomial regression with an exchangeable correlation matrix, adjusted for clustering by health facility. Secondary outcomes were calculated in a similar model. We conducted all analyses in SAS v9.4 (IBM, Armonk, NY, USA).

In addition to the composite primary outcome, ITT analysis on the appropriate treatment of malaria, pneumonia, and diarrhoea was performed separately. The study was not powered to show differences in the appropriate treatment of each of these conditions, but a disaggregated analysis by condition was important to help define the burden of disease and the relative success in appropriate treatment for each condition.

### Ethics, consent and permissions

We obtained approvals from both the Boston University and ERES Converge IRBs, and clearance from the Zambia National Health Research Authority. We also received approval from the Chipata and Chadiza district health offices. Informed consent was obtained from all study participants and study participation was voluntary. Three categories of study participants were required to provide informed consent; supervisors, CHWs and caregivers. For all the three categories, detailed written consent forms were prepared explaining the purpose of the study, participant roles in the study and that participation was voluntary and that they were free to decline. For supervisors and CHWs, consent forms were prepared in English while consent forms for caregivers were translated in the local language. For all these categories, the consent process involved reading the contents of the consent form to participants and allowing them to ask questions and providing clarifications. After providing clarifications, participants were asked to either consent by signing the consent forms or decline to sign. The consent form for caregivers had a provision for signing (if literate) or using a thumbprint for those who were not able to sign using a pen. As stated earlier, we had to increase the sample size during the study and the increase in sample size was approved by the local (ERES Converge) and the Boston University IRBs.

## RESULTS

### Recruitment

**Figure 1:**
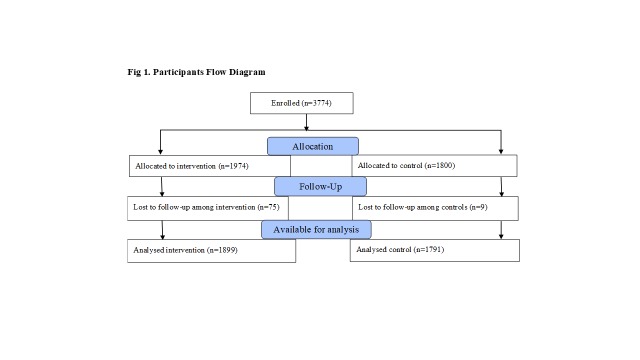
Participants’ flow diagram.

We enrolled 3774 study participants between February and July 2016, and analysed data on 3690 children under five years with malaria, diarrhoea, or pneumonia. **Figure 1** summarizes the numbers allocated and analysed by study arm.

### Baseline socio-demographic characteristics of study participants

We collected a limited amount of demographic information on the children: age and sex (**Table 1**). Approximately one third of the children in our sample were aged between 36 and 59 months. The intervention and control groups were comparable in terms of age and gender of children.

**Table 1:** Baseline demographic characteristics of children 2-59 mo assessed for treatment by CHWs by study arm (n, %)

Variable	Intervention	Control	All subjects
Number of patients	1899	1791	3690
**Age group (months):**			
2-11	22.9 (435)	23.3 (417)	23.1 (852)
12-23	25.1 (477)	24.3 (436)	24.7 (913)
24-35	19.5 (371)	20.1 (360)	19.8 (731)
36-59	32.4 (616)	32.3 (578)	32.4 (1,194)
**Sex:**			
Male	50.1 (952)	50.3 (900)	50.2 (1,852)
Female	45.4 (861)	46.2 (828)	45.8 (1,689)
Missing sex	4.5 (86)	3.5 (63)	4.0 (149)

We trained 80 CHWs, but 69 participated in the study (34 intervention; 35 control). Out of the 80 trained CHWs, 1 with serious learning difficulties was dropped, all 79 consented, but only 69 were available and participated when data collection finally started (10 dropouts). Consenting exercise took place on the last day of training. CHWs consisted of more men than women (67% vs 33%); most were married (87%) and aged between 35 and 49 years. Seventy percent of the CHWs had attained secondary level of education. The vast majority of CHWs reported farming as their primary occupation (85%). Demographic characteristics were similar between the two study arms except for ethnic group and religion.

We trained 40 supervisors, and all 40 consented, 6 dropped out before data collection started; most of them were transferred from their work stations and some went for further studies. Thirty-four supervisors therefore participated in the study when data collection started (14 intervention; 20 control). Consent was obtained on the last day of training. Approximately three-quarters of the supervisors were male, only 50% were married, and they were younger than the CHWs they supervised. More than 50% of the supervisors were nurses, and the rest comprised of midwives, clinical officers and environmental health technicians. There was also approximate balance of children’s demographic characteristics between the two study arms except regarding age, which was slightly higher in the control group.

### Appropriate treatment by study arm

In the intervention group, 65.9% of children 2-59 months old received appropriate care for iCCM conditions, compared to 63.3% in the control group (PR = 1.04; 95% CI = 0.94, 1.16) (**Table 2**).

**Table 2:** Proportion of children 2-59 mo of age given appropriate treatment by CHWs by study arm

Variable	Intervention, % (n/N)	Control, % (n/N)	All subjects, % (n/N)	PR (95% CI)
**Appropriate treatment of malaria, diarrhoea, and/or pneumonia**	65.9 (1,252/1,899)	63.3 (1,134/1,791)	64.7 (2,386/3,690)	1.04 (0.94, 1.16)
**Appropriate treatment of individual illnesses**				
Malaria	85.7 (780/910)	87.7 (750/855)	86.7 (1530/1765)	0.97 (0.93, 1.02)
Diarrhoea:				
-Including zinc	40.4 (158/391)	47.2 (168/356)	43.6 (326/747)	0.91 (0.55, 1.49)
-Excluding zinc	87.2 (341/391)	93.3 (332/356)	90.1 (673/747)	0.93 (0.88, 0.99)
Pneumonia	52.3 (426/814)	45.4 (361/795)	48.9 (787/1,609)	1.21 (0.87, 1.67)

CHW – community health worker, PR – prevalence ratio, CI – confidence interval

More CHWs appropriately prescribed amoxicillin for pneumonia in the intervention arm compared to the control but the difference was not statistically significant (PR = 1.21; 95% CI = 0.87, 1.67). **Table 2** shows the results of the primary variable, depicting high levels of appropriate treatment for malaria (86.7%, n = 1530) and diarrhoea excluding zinc (90.1%, n = 673) among intervention and control groups combined. The appropriate treatment of diarrhoea including zinc was low, although this may have been due to limited availability of this adjunct to oral rehydration therapy.

### Supervision, mentorship, and supply chain management

Monthly supervision and mentorship coverage was 18% higher in the intervention than the control arm but was not statistically significant (PR = 1.18; 95% CI = 0.95, 1.47) (**Table 3**).

**Table 3:** Monthly supervision and mentorship contacts by study arm*

Variable	Intervention	Control	All	PR (95% CI)
	N = 160, % (n)	N = 150, % (n)	N = 310, % (n)	
**Received supervision & mentorship in the last month**	61.3 (98)	52.0 (78)	56.8 (176)	1.18 (0.95, 1.47)
Cadre of supervisor	N = 98	N = 78	N = 176	
Health facility staff	96.9 (95)	89.7 (70)	93.8 (165)	
DHMT	3.1 (3)	7.7 (6)	5.1 (9)	
CHA	0.0 (0)	0.0 (0)	0.0 (0)	
Other	0.0 (0)	2.6 (2)	1.1 (2)	
Place of supervision	N = 98	N = 78	N = 176	
At the CHP	27.8 (27)	26.9 (21)	27.4 (48)	
At the health facility	68.0 (66)	53.8 (42)	61.7 (108)	
Other	4.1 (4)	17.9 (14)	10.3 (18)	
**Receipt of SMS reminder by CHW on use of sick child recording form and submission of weekly report**	N = 160, % (n)			
Reported SMS	39.4 (63)	N/A	N/A	
Confirmed SMS	42.9 (27)	N/A	N/A	
**No. of CHWs sending weekly disease management reports using mobile phone**	% (n/N)			
Weekly disease management reports	45.0 (72/160)	N/A	N/A	
Weekly drug reports	42.8 (68/159)	N/A	N/A	
Weekly new born care reports	36.5 (58/159)	N/A	N/A	
**No. of CHWs receiving feedback by phone from supervisors on patients referred**	N = 160, % (n)			
Reported SMS	13.1 (21/160)	N/A	N/A	
Confirmed SMS	47.6 (10/21)	N/A	N/A	

PR –prevalence ratio DHMT – district health management team, CHA-community health assistant, CHP – community health post, CHW – community health worker

*N = Total number of expected supervision and mentorship contacts.

Health facility staff carried out most of the supervision (93.8%; n = 165) and supervision and mentorship were mainly carried out at the health facility (61.7%; n = 108). The reporting rates for weekly disease management and drug supply management were 45.0% and 42.8% respectively in the intervention arm (**Table 3**). Forty-three percent (n = 27) of CHWs in the intervention arm reported receiving an SMS reminder on the use of the sick child recording form and submission of weekly reports. About half of CHWs referring patients to health posts reported receiving feedback from their supervisors on patient outcomes (47.6%, 10/21).

In terms of supply chain management, the overall prevalence of submission of reports and requisitions by CHWs by mobile phone was 27.9% (n = 67). There was no difference between the intervention and control arms in the availability of iCCM commodities on the day of the survey visit or reported stockouts during the month before the survey (**Table 4**). More CHWs in the intervention arm reported receipt of iCCM commodities that they ordered than CHWs in the control arm with prevalence ratios ranging from 2.62 for mRDTs (95% CI 1.37, 5.04) to 3.59 for amoxicillin (95% CI 1.79, 7.20).

**Table 4:** Proportion of CHWs reporting monthly commodity availability by study arm*

Variable	Intervention, N = 240, % (n)	Control, N = 241, % (n)	All subjects, N = 481, % (n)	PR (95% CI)
**Submit drugs report and requisition form using phone:**				
Submitted report	27.9 (67)	0.8 (2)	14.3 (69)	33.6 (8.8, 128.5)
**iCCM commodities available on day of visit:**				
Artemether-lumefantrine	50.4 (121)	45.2 (109)	47.8 (230)	1.12 (0.82, 1.52)
mRDTs	44.2 (106)	39.8 (96)	42.0 (202)	1.11 (0.80, 1.55)
Amoxicillin	52.5 (126)	48.1 (116)	50.3 (242)	1.09 (0.83, 1.43)
ORS	60.4 (145)	55.2 (133)	57.8 (278)	1.10 (0.59, 1.70)
Zinc	26.7 (64)	26.6 (24)	26.6 (128)	1.01 (0.59, 1.70)
All above	12.9 (31)	13.3 (32)	13.1 (63)	0.98 (0.51, 1.86)
**Stockouts of iCCM commodities past month before visit:**				
Artemether-lumefantrine	30.8 (74)	33.2 (80)	32.0 (154)	0.93 (0.69, 1.26)
mRDTs	35.4 (85)	35.3 (85)	35.3 (170)	1.00 (0.77, 1.31)
Amoxicillin	24.2 (58)	22.4 (54)	23.3 (112)	1.08 (0.71, 1.65)
ORS	11.7 (28)	11.2 (27)	11.4 (55)	1.04 (0.64, 1.68)
Zinc	44.6 (107)	40.2 (97)	42.4 (204)	1.11 (0.75, 1.64)
**Received supplies of iCCM commodities ordered:**				
Artemether-lumefantrine	37.5 (90)	13.3 (32)	25.4 (122)	2.82 (1.50, 5.30)
mRDTs	31.7 (76)	12.0 (29)	21.8 (105)	2.62 (1.37, 5.04)
Amoxicillin	35.8 (86)	10.0 (24)	22.9 (110)	3.59 (1.79, 7.20)
ORS	36.3 (87)	11.6 (28)	23.9 (115)	3.11 (1.70, 5.71)
Zinc	15.0 (36)	5.0 (12)	10.0 (48)	3.01 (1.29, 7.00)

PR – prevalence ratio, CI – confidence interval, ORS – oral rehydration salts, mRDT – malaria rapid diagnostic tests, iCCM – integrated community case management

* N = Total number of expected commodity reports submitted.

### CHWs illness classification of iCCM conditions by study arm

Both intervention and control CHWs displayed moderate levels of competence as measured by their ability to classify each of the three iCCM conditions (**Table 5**) in comparison to recorded symptoms.

**Table 5:** Comparison of illness classification of iCCM conditions by CHW and recorded diagnostic criteria by study arm

Variable	Intervention, N = 1899, % (n)	Control, N = 1791, % (n)	All subjects, N = 3690, % (n)
Classification by CHWs:			
Malaria	48.8 (927)	48.6 (970)	48.7 (1797)
Diarrhoea	20.0 (380)	19.4 (347)	19.7 (727)
Pneumonia	47.6 (904)	51.3 (918)	49.4 (1822)
Classification based on recorded diagnostic criteria			
Malaria	47.9 (910)	47.7 (855)	47.8 (1765)
Diarrhoea	20.6 (391)	19.9 (356)	20.2 (747)
Pneumonia	42.9 (814)	44.4 (795)	43.6 (1609)
Interoperator Agreement			Kappa statistic (95% CI)
Malaria			0.98 (0.97, 0.98)
Diarrhoea			0.93 (0.92, 0.95)
Pneumonia			0.80 (0.78, 0.82)

CHW – community health worker, CI – confidence interval

Overall, a comparison of illness classification by CHWs and classifications based on recorded presenting symptoms and signs indicate a kappa of 0.98 (95% CI = 0.97, 0.98), 0.93 (95% CI = 0.92, 0.95), and 0.80 (95% CI = 0.78, 0.82) for malaria, diarrhoea and pneumonia, respectively.

## DISCUSSION

The results of this study show a higher prevalence of supportive supervision in the intervention group compared to the control. This difference, however, was not statistically significant and did not translate into statistically significant increases in the appropriate treatment of malaria, diarrhoea, or pneumonia. We observed no effect of the intervention on the proportion of children appropriately treated for malaria, diarrhoea and pneumonia by CHWs. We also found that mHealth interventions were associated with a 2-3-fold non-significant increase in the proportion of CHWs that received supplies they had ordered, but there was no overall impact on stockouts of iCCM commodities. This is possibly attributable to the low coverage of the intervention at 61% supervision and 28% prevalence of report submission and requisition using mHealth technology. This low coverage may have been due to the short implementation period of 5.5 months.

CHWs have used mHealth technologies to advance a range of health care objectives globally, particularly for enhancement of their supportive supervision [32]. However, in order to have an impact on CHW performance, health service coverage, and ultimately health impact, as well as both frequency and quality of supervision are essential [33,34]. Although we did not identify a cut off point for the level of supervision required to have an impact on health outcomes, we are of the view that supervision coverage of 61% is too low and may therefore explain the reason for the observed low impact on appropriate treatment.

This study did not measure supervision quality; however, the mHealth platform was designed to permit supervisors from the District Health Office and central level health personnel with access privileges to be able to review supervisors’ activities. For example, the district iCCM focal point persons and the Principal IMCI Officer from MOH headquarters used the system to monitor iCCM activities in each district.

Some of the important features of the mHealth enhanced supervision and mentorship program were the feedback provided to the CHWs by supervisors on patients they received and mentorship reports the supervisors produced. Almost half (47.6%) of CHWs reported receiving feedback by phone from their supervisors on patients they referred.

This feedback not only provided an opportunity for CHWs to know the final outcome of the patients they referred but may also have acted as a motivating factor to the CHWs. Positive performance feedback has been shown to have a significant effect on health worker compliance with IMCI treatment guidelines [35] and on health worker performance [36,37]. The 21% increase in appropriate treatment for pneumonia by CHWs observed in this study, though not statistically significant, is clinically important to note as a potential benefit of mHealth technology on CHW performance, because it suggests that, with mHealth interventions, CHWs are able to provide appropriate treatment to one in every five more children with pneumonia than they currently do. Overall, appropriate treatment for pneumonia was low in this study, as only 48.9% of children with pneumonia were prescribed appropriate medication compared to 78% to 98.9% frequency of appropriate treatment reported in other studies conducted in Zambia [38,39]. The reasons for inappropriate treatment of pneumonia in this study include misdiagnosis and prescription of the wrong medication for those who were correctly diagnosed. More capacity building in the diagnosis and treatment of pneumonia by CHWs is therefore recommended.

Overall, the CHWs in this study in both control and intervention arms displayed high levels of competence in terms of correct classification of iCCM conditions and appropriate treatment for malaria and diarrhoea. This is mainly due to the fact that both groups had recently been trained in iCCM. However, they performed well only in two of the three iCCM conditions, most likely due to the fact that pneumonia is more complicated to diagnose and treat. These results are similar to those found by a recent demonstration project in Uganda [40]. In the Ugandan demonstration study, appropriate treatment for pneumonia was lowest at 88.2% vs 97.1% for fever and 92.4% for diarrhoea.

Apart from the effect on appropriate treatment for pneumonia, the mHealth intervention was associated with good response from health facilities on iCCM commodities ordered as CHWs in the mHealth supported study arm were more than 2-3 times likely to receive the commodities they ordered compared to the non-mHealth supported CHWs. There is evidence that mHealth technologies are associated with lower lead times for drug re-supply and lower mean drug stockout rates [41]. What remains unclear in our study is why there was no difference between the mHealth supported CHWs and the non-mHealth supported CHWs in terms of availability of iCCM commodities and stockouts, despite more CHWs in the intervention reporting that they received the commodities they ordered. Caseload analysis from a costing component of this study, to be reported in a separate publication, showed that the intervention CHWs saw a total of 9356 cases of the three iCCM conditions while the control CHWs saw a total of 6069 cases. This means that although intervention CHWs received the commodities they ordered most of these commodities were consumed by the higher number of children they attended to. In fact, commodities consumption data showed that the intervention CHWs dispensed 60% of the commodities.

### Limitations

This study has several limitations. As indicated above, insufficient time for full uptake of the intervention may have limited its impact. The study was powered to detect a difference of 15% in the appropriate treatment of malaria, diarrhoea, and pneumonia, and the observed difference in the study was only 2.6%; greater time to allow the intervention to proceed might have resulted in greater impact. However, available funds could not allow for a longer period of implementation. Lack of data on the frequency of paper-based reports and requisitions in the control arm makes it difficult to compare the two arms for this indicator (frequency of paper-based reports).

## CONCLUSION

This study was unable to determine whether using mHealth technology would strengthen supervision and supply chain management of iCCM commodities for community-level workers. The study was also not able to demonstrate a statically significant effect of mHealth enhanced iCCM on appropriate diagnosis and treatment for children with malaria, pneumonia, and diarrhoea in rural Zambia. Longer term longitudinal studies are required to determine the impact of mHealth enhanced iCCM on health outputs and outcomes.

## Additional material

Online Supplementary Document
