# Supplementary material for: Impact of mobile health-enhanced supportive supervision and supply chain management on appropriate integrated community case management of malaria, diarrhoea, and pneumonia in children 2-59 months: A cluster randomised trial in Eastern Province, Zambia
Source: J Glob Health. 2020 May 15;10(1):010425. doi: 10.7189/jogh.10.010425 (PMC7243069; doi:10.7189/jogh.10.010425)
Supplement: Online Supplementary Document [file jogh-10-010425-s001.pdf]

Table S1: Key variables, definitions and methods of collection

**Baseline form**	
Purpose of the instrument	
To extract child level data from the sick child recording form, including patient demographics, presenting symptoms, examination findings, and treatment given. The Sick Child Recording Form is a form that is routinely completed by a CHW each time they see a child with an iCCM condition and is retained at the health centre. For this study, data collectors extracted the information from a completed form. The form had information on the child and the caregiver's contact details.	
Key Information Collected	***Key Variables***
Self-reported information, signs and symptoms	Age of child<br>Sex of child<br>Duration of illness<br>Presenting complaints and danger signs.
Observed signs and symptoms	Respiratory rate<br>Presence of lower chest wall indrawing<br>Malaria RDT results
Classification of disease	Malaria, diarrhoea, and/or pneumonia
Treatment	Anti-malarial<br>Antibiotic<br>Zinc<br>ORS
**Day 5-7 follow-up form**	
Use of the instrument	
To collect data to measure study outcomes on all enrolled children. This form was administered to the caregiver 5-7 days after the child was seen by a CHW. The data collectors checked the date when the child was seen by a CHW and then followed up 5-7 days later. This information was collected through interviews with Caregivers.	
Key Information Collected	***Key Variables***
Demographic information on parents	Relationship of respondent to child<br>Age<br>Education<br>Employment<br>Marital status<br>Approximate monthly household income<br>Head of household<br>Number of household members
Information on child	Age of child<br>Sex of child<br>Breastfeeding status<br>Current status
Information about diagnosis and treatment	Initial complaints<br>Medicines prescribed

	Medication adherence<br>Additional care/treatment outside CHW<br>Additional symptoms<br>Current status
**Monthly activity form**	
Use of the instrument	
This form was administered to CHWs to collect data on supportive supervision and mentorship received the previous month.	
Key Information Collected	***Key Variables***
Supervision and mentoring	Did supervisory visit take place and, if so, what was the content of that visit?
Weekly SMS reporting	Which weekly reports did CHW send via SMS?
Supplies	Current availability of drugs and stockouts in the last month
Workload and patients seen	Number of patients seen by age group and diagnosis.
**CHW assessment form**	
Use of the Instrument	
This was administered to CHWs to collect their socio-demographic information, training, and work-related data.	
Key Information Collected	***Key Variables***
Socio-demographic information	Age<br>Sex<br>Education<br>Ethnic group<br>Languages<br>Religion<br>Marital status<br>Main occupation
CHW activities	Years worked<br>Hours work weekly<br>Services provide for children<br>Distance from CHP to CHC<br>Mode of transportation<br>Supervision in community<br>Paid for CHW work<br>Amount paid<br>Satisfaction<br>Motivators and demotivators
Training received as CHW	Types of training received in past 2 years

**Supervisor assessment form**	
Use of the instrument	
The form was administered to the supervisors of CHWs to collect their socio-demographic information, training, and work-related data.	
Key Information Collected	***Key Variables***
Socio demographic information	Age<br>Sex<br>Education<br>Ethnic group<br>Languages<br>Religion<br>Marital status<br>Occupation
Activities as CHW supervisor	Years worked as CHW supervisor<br>Hours work supervising weekly<br>Supervision services provide<br>Greatest distance to visit CHW<br>Mode of transportation<br>Paid for supervision visits<br>Amount paid<br>Satisfaction with work as supervisor
Training received for CHW supervisor	Types of training received in past 2 years

**Key Variables**	**Variable Definition**	**Data Collection Instrument**	**Use of the Instrument**
Self-reported information, signs and symptoms	Age of child<br>Sex of child<br>Duration of illness<br>Presenting complaints and danger signs.	Baseline form	To extract child level data from the sick child recording form, including patient demographics, presenting symptoms, examination findings, and treatment given. The Sick Child Recording Form is a form that is routinely completed by a CHW each time they see a child with an iCCM condition and is retained at the health centre. For this study, data collectors extracted the information from a completed form. The form had information on the child and the caregiver’s contact details.
Observed signs and symptoms	Respiratory rate<br>Presence of Lower chest wall indrawing<br>Malaria RDT results		
Classification of	Malaria, Diarrhoea,		

disease	and/or Pneumonia		
Treatment	Anti-malarial<br>Antibiotic<br>Zinc<br>ORS		
Demographic information on parents	Relationship of respondent to child<br>Age<br>Education<br>Employment<br>Marital status<br>Approximate monthly household income<br>Head of household<br>Number of household members	Day 5-7 follow-up form	To collect data to measure study outcomes on all enrolled children. This form was administered to the caregiver 5-7 days after the child was seen by a CHW. The data collectors checked the date when the child was seen by a CHW and then followed up 5-7 days later. This information was collected through interviews with Caregivers.
Information on child	Age of child<br>Sex of child<br>Breastfeeding status<br>Current status		
Information about diagnosis and treatment	Initial complaints<br>Medicines prescribed<br>Medication adherence<br>Additional care/treatment outside CHW<br>Additional symptoms<br>Current status		
Supervision and mentoring	Did supervisory visit take place and, if so, what was the content of that visit?	Monthly activity form	This form was administered to CHWs to collect data on supportive supervision and mentorship received the previous month.
Weekly SMS reporting	Which weekly reports did CHW send via SMS?		

Supplies	Current availability of drugs and stockouts in the last month		
Workload and patients seen	Number of patients seen by age group and diagnosis.		
Socio demographic information	Age<br>Sex<br>Education<br>Ethnic group<br>Languages<br>Religion<br>Marital status<br>Main occupation	CHW assessment form	This was administered to CHWs to collect their socio-demographic information, training, and work-related data.
Activities as CHW	Years worked<br>Hours work weekly<br>Services provide for children<br>Distance from CHP to CHC<br>Mode of transportation<br>Supervision in community<br>Paid for CHW work<br>Amount paid<br>Satisfaction<br>Motivators and demotivators		
Training received as CHW	Types of training received in past 2 years		
Socio demographic information	Age<br>Sex<br>Education<br>Ethnic group<br>Languages<br>Religion<br>Marital status	Supervisor assessment form	The form was administered to the supervisors of CHWs to collect their socio-demographic information, training, and work-related data.

	Main occupation		
Activities as CHW supervisor	Years worked as CHW supervisor<br>Hours work supervising weekly<br>Supervision services provide<br>Greatest distance to visit CHW<br>Mode of transportation<br>Paid for supervision visits<br>Amount paid<br>Satisfaction with work as supervisor		
Training received for CHW supervisor	Types of training received in past 2 years		
